# Influence of high density lipoprotein cholesterol levels on circulating monocytic angiogenic cells functions in individuals with type 2 diabetes mellitus

**DOI:** 10.1186/s12933-018-0720-1

**Published:** 2018-06-05

**Authors:** Daniela Lucchesi, Simona Georgiana Popa, Veronica Sancho, Laura Giusti, Monia Garofolo, Giuseppe Daniele, Laura Pucci, Roberto Miccoli, Giuseppe Penno, Stefano Del Prato

**Affiliations:** 1 Section of Diabetes and Metabolic Disease, Department of Clinical and Experimental Medicine, University of Pisa and Azienda Ospedaliero-Universitaria Pisana, Via Paradisa, 2, 56124 Pisa, Italy; 2 Diabetes, Nutrition and Metabolic Diseases, University of Medicine and Pharmacy of Craiova, Craiova, Romania; 3 Institute of Agricultural Biology and Biotechnology, National Research Council (CNR), Pisa, Italy

**Keywords:** Type 2 diabetes mellitus, Endothelial progenitor cells, Monocytic angiogenic cells, High-density lipoprotein cholesterol, Endothelial function

## Abstract

**Background:**

High-density lipoproteins (HDLs) can exert anti-atherogenic effects. On top of removing excess cholesterol through reverse cholesterol transport, HDLs play beneficial actions on endothelial function and integrity. In particular, HDLs are strong determinant of endothelial progenitor cells (EPCs) number and function. To gain further insights into such an effect we characterized in vitro functionality of circulating “early” EPCs obtained from 60 type 2 diabetes individuals with low HDL-cholesterol (HDL-C) and 59 with high HDL-C levels.

**Methods:**

After an overnight fast, venous blood was drawn in EDTA tubes and processed within 2-h from sampling. Peripheral blood mononuclear cells were isolated and plated on fibronectin coated culture dishes; after 3 days culture, adherent cells positive for Dil-ac-LDL/Lectin dual fluorescent staining were identified as monocytic angiogenic cells (MACs). After 5–7 days culture in EBM-2 medium, adherent cells were evaluated for viability/proliferation (MTT assay), senescence (beta-galactosidase activity detection), migration (modified Boyden chamber using VEGF as chemoattractant), adhesion capacity (on fibronectin-coated culture dishes) and ROS production (ROS-sensitive fluorescent probe CM-H_2_DCFDA).

**Results:**

MACs obtained from diabetic individuals with high HDL-C had 23% higher viability compared to low HDL-C (111.6 ± 32.7% vs. 90.5 ± 28.6% optical density; p = 0.002). H_2_O_2_ exposure impaired MACs viability to a similar extent in both groups (109.2 ± 31.7% vs. 74.5 ± 40.8% in high HDL-C, p < 0.0001; 88.3 ± 25.5% vs. 72.3 ± 22.5% in low-HDL, p = 0.004). MACs senescence was comparable in the two groups (102.7 ± 29.8% vs. 99.2 ± 27.8%; p = 0.703) and was only slightly modified by exposure to H_2_O_2_. There was no difference in the MACs migration capacity between the two groups (91.3 ± 34.2% vs. 108.7 ± 39.5%; p = 0.111), as well as in MACs adhesion capacity (105.2 ± 32.7% vs. 94.1 ± 26.1%; p = 0.223). Finally, ROS production was slightly thought not significantly higher in MACs from type 2 diabetes individuals with low- than high-HDL. After stratification of HDL-C levels into quartiles, viability (p < 0.0001) and adhesion (p = 0.044) were higher in Q4 than in Q1–Q3. In logistic regression analysis, HDL-C was correlated to MACs viability and adhesion independently of HbA1c or BMI, respectively.

**Conclusions:**

Our data suggest that in type 2 diabetes subjects, HDL-cholesterol is an independent determinant of circulating MACs functional capacities—mainly viability, to a lesser extent adhesion—likely contributing also through this mechanism to cardiovascular protection even in type 2 diabetes.

## Background

Epidemiological and clinical studies indicate that plasma levels of high-density lipoproteins (HDLs) cholesterol (HDL-C) are associated with lower risk of coronary artery disease. Indeed, HDL particles are known to exert a broad spectrum of anti-atherogenic properties [1]: they remove cholesterol excess through reverse cholesterol transport from peripheral tissue to the liver, inhibit lipid oxidation, exert anti-inflammatory, anti-thrombotic, anti-proteolytic and anti-apoptotic effects, inhibit intracellular oxidative stress, and restore endothelial dysfunction [2]. HDLs also contribute to nitric oxide bioavailability to ensure vasodilation [3] and facilitate repair of endothelial injuries [1, 2].

Endothelial repair is largely dependent on recruitment of circulating endothelial progenitor cells (EPCs) in the areas of intimal injury and activation of quiescent tissue-resident endothelial cells or resident EPCs via the release of paracrine factors [2]. Resident endothelial cells/EPCs and endothelial cell-derived microparticles (EMPs) can provide direct sources of paracrine factors with angiogenic and angio-protective properties [2, 4].

HDLs exert a potent effect on the number and function of circulating EPCs [5–8]. In vitro, human HDLs enhance differentiation of mononuclear cells into early EPCs, inhibit their apoptosis [9, 10], increase their migratory capacity, and propensity to adhesion [5]. In a model of apolipoprotein E-deficient (apoE^−/−^) mice, intravenous infusion of reconstituted HDLs (rHDLs) more than doubled the number of EPCs engrafted in the aortic endothelium [11]. In EPCs isolated from the bone marrow of hypercholesterolemic rats, HDLs promoted EPCs proliferation, migration, and tube formation [12]. In a low-density lipoprotein (LDL) receptor deficient (LDLr^−/−^) mice, lipid lowering or HDLs resulted in a two-fold increase of circulating EPCs and improved function as assessed by ex vivo EPCs migration and adhesion [13]. Finally, in a recent study, impaired viability of late outgrowth EPCs (generated from human peripheral mononuclear cells, PBMCs) induced by oxidized LDL (oxLDL) was reversed by HDLs in a dose dependent manner. Of note, in the absence of oxLDL, low HDL concentration enhanced EPCs tube formation, while moderate to high concentrations paradoxically enhanced EPCs senescence and impaired tube formation [14].

This already complex pattern of interaction between HDL particles and the endothelium becomes even more complicated in the diabetic condition [3, 15, 16] as both HDLs [17] and EPCs [18, 19] from diabetic patients have been found to be dysfunctional. Changes in HDLs metabolism related to insulin resistance, glycation and depletion of apolipoprotein A-1 (apoA-1), modifications of other HDL-associated proteins such as paraoxonase-1 (PON-1) or oxidation of components of the HDL particles, and alterations of HDL proteome induced by chronic inflammation, all contribute to HDL dysfunction in diabetes [17]. Compared with healthy subjects, HDLs from individuals with type 2 diabetes show impaired in vitro endothelial-protective effects in the aortic ring segments from mice [20], while infusion of rHDLs in type 2 diabetes patients restored serotonin induced vasodilation [21]. In particular, HDLs from healthy, but not from diabetic subjects, promoted in vivo endothelial repair by early EPCs obtained from patients with diabetes in a model of carotid injury in mice [20]. Furthermore, in diabetic subjects the number of circulating EPCs is reduced and their function altered even in the absence of diabetic complications [18, 19].

Within this scenario, we now report results of a study that evaluated the relationship between HDL-cholesterol levels and ex vivo functional properties of monocytic angiogenic cells (MACs), originally defined as “early” EPCs, isolated from individuals with type 2 diabetes.

## Methods

### Patients characteristics and study design

A total of 119 men and women with type 2 diabetes for more than 12 months and on stable anti-diabetic treatment regimen (oral anti-diabetic medications and/or long acting or short acting insulin) for the prior 3 months were consecutively recruited at the Diabetes Unit of the Department of Clinical and Experimental Medicine of the University of Pisa. Pregnant or breastfeeding women, individuals of non European descent, subjects with type 1 diabetes, those with advanced, sight-threatening diabetic retinopathy or chronic kidney disease (CKD) stages ≥ 3b, as well those with poor glycemic control, defined as HbA1c ≥ 9.0% as measured at the enrollment visit, were excluded. Further exclusion criteria were body mass index (BMI) ≥ 35 kg/m^2^, severe uncontrolled hypertension defined as systolic blood pressure (BP) ≥ 180 mmHg and/or diastolic BP ≥ 110 mmHg, sign of acute illness or infection, significant hepatic disease, chronic inflammatory disease, immunological and myeloproliferative diseases or other malignancy, and any major cardiovascular event within 3 months of entering in the study. All patients underwent a structured interview in order to collect information about the onset of diabetes, its duration, smoking habits, current anti-diabetic treatment, and BP- and lipid-lowering therapies [22]. Body weight and height were assessed and BMI calculated; waist circumference was measured at midway between the costal margins and the iliac crests. BP was measured after 5-min rest while seated and the average of two consecutive measurements obtained about 5-min apart was calculated. Hypertension was defined as systolic BP > 140 mmHg and/or diastolic BP > 80 mmHg and/or use of any antihypertensive medication.

In all subjects a venous blood specimen was drawn after an overnight fast for determination of HbA1c (high-performance liquid chromatography using DCCT-aligned methods) [23], serum creatinine, glucose, total-, HDL-, and LDL-cholesterol (Friedewald formula), triacylglycerol, alanine aminotransferase (ALT), aspartate aminotransferase (AST), gamma-glutamyltransferase (GGT), uric acid, fibrinogen and blood cell count using standard laboratory methods. A first-voided urine sample was also collected for determination albumin (BNII; Dade Behring Diagnostic, Marburg, Germany) and creatinine (modified Jaffé reaction). Urinary samples with abnormal sediments on routine analysis were excluded. Finally, all patients underwent a screening for diabetic complications as detailed elsewhere [22]. Briefly, diabetic retinopathy was assessed by retinal photography and its severity classified according to the Global Diabetic Retinopathy Project Group criteria [24]; patients with advanced, sight-threatening retinopathy (severe non-proliferative, proliferative, maculopathy or blindness) were excluded. Based on ACR (albumin-to-creatinine ratio) values, UAE (urinary albumin excretion) categories were defined as normoalbuminuria (< 30 mg/g, < 3.4 mg/mmol), microalbuminuria or “moderately increased albuminuria” (30–299 mg/g, 3.4–34 mg/mmol) and macroalbuminuria or “severely increased albuminuria” (≥ 300 mg/g, ≥ 34 mg/mmol). Estimated glomerular filtration rate (eGFR) was calculated by the CKD-EPI (Chronic Kidney Disease Epidemiology Collaboration) equation [25] and subjects with eGFR < 45 ml/min/1.73 m^2^ (CKD stages ≥ 3b) were excluded. Diabetic neuropathy was assessed by a validated questionnaire, by knee and ankle reflexes and by measurement of vibration perception threshold (VPT) [22]. Finally, previous cardiovascular diseases (CVD) were determined based on medical history by recording all documented major acute CVD events and revascularization procedures. Peripheral vascular disease was also assessed by search of femoral and foot pulses, and measurement of ankle/brachial pressure ratio [22].

Patients were divided in subjects with low [≤ 40/50 mg/dl (≤ 1.034/1.293 mmol/l) in men/women] and high HDL-C levels [> 40/50 mg/dl (> 1.034/1.293 mmol/l) in men/women]. The main characteristics of the two study groups are given in Table 1. In all these subjects, a 50 ml fasting blood sample was drawn in EDTA-containing tubes and processed within 2 h from collection for culture and assessment of MACs functional properties (see below).

**Table 1:** The main anthropometric and clinical characteristics of the study cohort

	All subjects	High HDL HDL > 40/50 mg/dl (M/F)	Low HDL HDL ≤ 40/50 mg/dl (M/F)	*p*
N. (%)	119	59 (49.6%)	60 (50.4%)	
Age, years	63.6 ± 7.9	65.0 ± 8.4	62.1 ± 7.2	0.047
Duration of diabetes, years	11.8 ± 9.8	13.7 ± 10.7	9.8 ± 8.5	0.031
Sex (M/F), n (%)	69/50 (58/42)	25/34 (42/58)	44/16 (73/27)	0.001
BMI, kg/m^2^	28.8 ± 5.4	27.2 ± 5.8	30.4 ± 4.4	0.001
BMI categories (< 25, 25–30, > 30 kg/m^2^), n (%)	27/54/38 (22.7/45.4/31.9)	22/27/10 (37.3/45.8/16.9)	5/27/28 (8.3/45.0/46.7)	0.0001
Waist circumference, cm	102.4 ± 13.3	97.6 ± 14.0	107.2 ± 10.7	0.0001
Systolic BP, mmHg	145.6 ± 17.1	145.2 ± 15.8	146.0 ± 18.5	0.813
Diastolic BP, mmHg	79.5 ± 9.1	77.1 ± 8.9	81.9 ± 8.6	0.004
Hypertension, n (%)	90 (75.6)	40 (67.8)	50 (83.3)	0.048
Fasting glucose, mg/dl (mmol/l)	146.4 ± 35.4 (8.13 ± 1.97)	147.1 ± 34.7 (8.17 ± 1.93)	145.6 ± 36.3 (8.09 ± 2.02)	0.814
HbA1c, % (mmol/mol)	7.27 ± 0.88 (56.0 ± 9.6)	7.24 ± 0.81 (55.6 ± 8.8)	7.31 ± 0.95 (56.3 ± 10.4)	0.683
Total cholesterol, mg/dl (mmol/l)	181.2 ± 33.4 (4.68 ± 0.86)	191.3 ± 34.2 (4.94 ± 0.89)	171.2 ± 29.6 (4.43 ± 0.77)	0.001
LDL cholesterol, mg/dl (mmol/l)	101.1 ± 27.8 (2.62 ± 0.72)	100.9 ± 29.0 (2.61 ± 0.75)	101.4 ± 26.8 (2.62 ± 0.69)	0.930
HDL cholesterol, mg/dl (mmol/l)	53.3 ± 42.0 (1.38 ± 0.58)	73.5 ± 13.0 (1.90 ± 0.34)	33.5 ± 5.1 (0.86 ± 0.13)	–
Non-HDL cholesterol, mg/dl (mmol/l)	127.8 ± 31.9 (3.31 ± 0.83)	117.8 ± 31.6 (3.05 ± 0.82)	137.8 ± 29.3 (3.56 ± 0.76)	0.001
Triacylglycerol, mg/dl [mmol/l]	124.0 (75.0–194.0) [1.40 (0.85–2.19)]	81.0 (59.0–120.0) [0.92 (0.67–1.36)]	185.5 (131.2–237.7) [2.10 (1.48–2.69)]	0.0001
Creatinine, mg/dl (µmol/l)	0.90 ± 0.28 (79.6 ± 24.8)	0.80 ± 0.17 (70.8 ± 14.8)	1.00 ± 0.33 (88.4 ± 29.3)	0.0001
Uric acid, mg/dl (µmol/l)	5.35 ± 1.46 (318.2 ± 86.8)	4.83 ± 1.27 (287.2 ± 75.5)	5.88 ± 1.46 (349.7 ± 86.7)	0.0001
Albumin to creatinine ratio, mg/g	7.3 (3.9–18.8)	7.4 (4.6–13.7)	6.1 (3.4–36.4)	0.457
A/C ratio categories: < 30, 30–300, > 300 mg/g; n (%)	95/20/4 (79.8/16.8/3.4)	50/7/2 (84.7/11.9/3.4)	45/13/2 (75.0/21.7/3.3)	0.358
eGFR, CKD-EPI, ml/min/1.73 m^2^	82.5 ± 18.3	85.8 ± 14.7	79.2 ± 20.9	0.052
AST, U/L	22.0 ± 14.9	20.8 ± 11.6	23.2 ± 17.7	0.377
ALT, U/L	23.7 ± 14.3	21.2 ± 12.6	26.1 ± 15.6	0.063
GGT, U/L	32.6 ± 29.4	33.9 ± 33.6	31.2 ± 24.9	0.624
Smoking habits: no smokers, ex-smokers, current smokers, n (%)	63/33/23 (52.9/27.7/19.3)	39/16/4 (66.1/27.1/6.8)	24/17/19 (40.0/28.3/31.7)	0.001
Glucose lowering treatments				
Metformin, n (%)	97 (81.5)	48 (81.4)	49 (81.7)	0.965
Sulphonilureas or glinides, n (%)	34 (28.6)	17 (28.8)	17 (28.3)	0.887
Thiazolidinediones, n (%)	6 (5.0)	4 (6.8)	2 (3.3)	0.390
DPP4 inhibitors, n (%)	36 (30.3)	15 (25.4)	21 (35.0)	0.256
GLP-1 receptor agonists, n (%)	11 (9.2)	3 (5.1)	8 (13.3)	0.120
Insulin, n (%)	35 (29.4)	19 (32.2)	16 (26.7)	0.507
BP-lowering treatments, n (%)	84 (70.6)	36 (61.0)	48 (80.0)	0.023
RAAS inhibitors, n (%)	70 (58.8)	32 (54.2)	38 (63.3)	0.313
Lipid-lowering treatments, n (%)	58 (48.7)	30 (50.8)	28 (46.7)	0.648
Non advanced retinopathy, n (%)	18 (15.1)	7 (11.9)	11 (18.3)	0.325
Major acute cardiovascular events (MACE), n (%)	21 (17.6)	7 (11.9)	14 (23.3)	0.101

The Ethics Committee of the University of Pisa approved the study protocol and written informed consent was obtained from all participants before any study procedure.

### Cell culture

MACs were cultured according to previously described techniques [26]. PBMCs were isolated from blood of each individual with type 2 diabetes by Biocoll (Biochrom AG; density = 1.077 g/ml) density-gradient centrifugation. Total PBMCs were seeded on 2 µg/cm^2^ fibronectin coated culture dishes (BD Falcon™) or on Lab-Tek ^®^II chamber slides system (Sigma-Aldrich Ltd, Poole, Dorset, UK) after red cell lyses. After isolation, cells were cultured in endothelial basal medium (EBM-2, Lonza Sales AG, Basel, Switzerland) supplemented with EGM-2-MV-SingleQuots containing human endothelial growth factor (EGF), hydrocortisone, insulin-like growth factor-1 (IGF-1), fibroblast growth factor (FGF), vascular endothelium growth factor (VEGF), ascorbic acid, antibiotics and 5% fetal bovine serum (FBS, Lonza Sales AG). After 3 days of culture, non-adherent cells were discarded by washing with phosphate buffered saline (PBS) and the culture medium was replenished daily. On day 5, adherent cells displaying an elongated spindle-shaped morphology were identified as MACs.

### MACs characterization

**Fig. 1:**
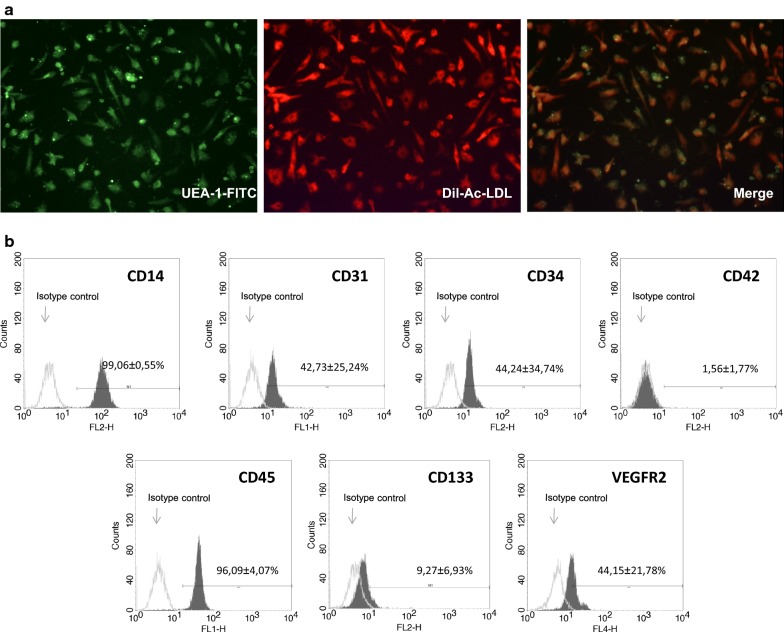
**a** MACs phenotype characterization by double staining with DiI-Ac-LDL uptake (on the left; red) and lectin UEA-1-FITC binding (in the middle, green). Merged images showed DiI-Ac-LDL/lectin double-positive MACs (on the right, yellow) (magnification 20×). **b** FACS quantification of the cell surface markers in MACs. The picture shows results (expression of each surface marker; mean ± SD) typically obtained from six separate experiments. Isotype controls are shown. CD14 and CD45 positivity clearly support the monocytic nature of the cells we have obtained. Growing body of evidence suggests that MACs closely resemble to M2-like macrophages which are characterized by anti-inflammatory features as well as to play pro-angiogenic functions [27]. Observations on surface expression of CD34, which may be lost—though not necessarily—during culture, are conflicting. In our study, flow cytometric analysis showed that cells were strongly positive for CD14 and CD45 with weaker expression of the hematopoietic lineage CD34 (which declines through cell culture passages). Our cells also expressed monocyte markers associated with endothelial cell features such as vascular endothelial growth factor receptor-2 (VEGFR2, also known as KDR) and platelet endothelial cell adhesion molecule-1 (PECAM-1, also known as CD31), suggesting MACs could be considered a sort of educated monocytes [28]. Several groups have reported the coexpression of endothelial markers by these cells. It has been suggested that the detection of endothelial markers might results from contamination with microparticles deriving from other elements in the culture (i.e. platelets) or by passive transfer of platelet microparticles containing CD31 leading to false-positive events by FACS quantification. Testing for CD42 (which, in our hands, was negative) allowed us to exclude contamination with and/or passive transfer of microparticles

MACs (“early” EPCs) were characterized for the uptake of 1,10-dioctadecyl-3,3,3,3-tetramethylindocarbo-cyanine-labeled acetylated low-density lipoprotein (DiI-ac-LDL) and for lectin binding. The staining was performed incubating cells with 10 µg/ml of DiI-ac-LDL (Invitrogen, Life Technologies Ltd, Paislet, UK) for 2 h at 37 °C in dark conditions. Cells were fixed in 4% paraformaldehyde for 30 min and counterstained with 1 mg/ml FITC-labelled lectin from *Ulex europaeus* (Sigma-Aldrich Ltd) for 2 h at 37 °C in dark conditions. Stained cells were observed by a fluorescence microscope and double positive DiI-ac-LDL/Lectin cells were identified as MACs. To evaluate the immunophenotype of MACs, adherent cells were detached with trypsin–EDTA and 5 × 10^5^ cell/tube were incubated with anti-human CD34-PE (BD Biosciences), CD133-PE (Miltenyi Biotec), VEGFR-2-Alexa Fluor 647 (BioLegend), CD31-FITC (BD Biosciences), CD45-FITC (BD Biosciences) and CD42-PE (BD Biosciences) for 30 min in dark conditions at 4 °C. Isotype control antibodies were used to set baseline fluorescence levels. The labeled cells were analyzed on a FACS-Calibur Instrument (BD Biosciences), acquiring 2 × 10^4^ events for each analysis. The flow cytometric analysis was repeated six times. After a 5 days culture under standard conditions, MACs resulted in an adherent population consisting of cells that showed elongated with a spindle-like shape and exhibited double positivity for Ac-LDL and lectin binding (UEA-1-FITC) as established by fluorescent microscope analysis (Fig. 1a). MACs phenotype was confirmed by the expression of main endothelial cell surface markers: CD14 (99.06 ± 0.55%), CD31 (42.73 ± 25.24%), CD34 (44.24 ± 34.74%), CD42 (1.56 ± 1.77%), CD45 (96.09 ± 4.07%), CD133 (9.27 ± 6.93%) and VEGFR-2 (44.15 ± 21.78%) (Fig. 1b).

### MACs viability

Viability of the cultured MACs was determined in cells obtained from 48 low-HDL and 40 high-HDL subjects by using the MTT assay. MTT (3-(4,5-Dimethylthiazol-2-yl)-2,5-diphenyltetrazolium bromide) measures mitochondrial activity in living cells. Briefly, after 5 days of culture MACs were incubated with MTT (Sigma, St. Louis, USA) (1 mg/ml) for 3 h at 37 °C, 5%/95% CO_2_/O_2_. Upon incubation, the medium was removed and the cells were solubilized in 10% DMSO/90% Isopropanol. Then, the amount of the dye released from the cells was quantified by measuring the optical density at 540 nm (reference wavelength: 620 nm) with a multiplate reader (Multiskan EX, THERMO). The optical density is directly correlated with the amount of metabolically active cells. To test the effect of an oxidative stress condition on MACs viability, H_2_O_2_ (1 mM, 1 h) was added to the culture medium.

### MACs adhesion to matrix molecules

Adhesion capacity was determined in MACs from 21 low-HDL and 24 high-HDL. To this purpose MACs were washed with PBS, and gently detached with 0.25% trypsin/EDTA. After centrifugation and re-suspension, equal cell numbers (50,000 cells/well) were seeded on fibronectin coated 96-well microplates, and incubated for 30 min at 37 °C, 5% CO_2_. The cells were fixed in 4% paraformaldehyde and then incubated with 0.25% crystal violet for 30 min; therefore, excess dye was removed by several washes with PBS and the dye absorbed by adherent cell nuclei was extracted with 33% AcOH. The amount of the dye released from the cells was quantified by measuring the optical density at 540 nm.

### MACs senescence

Senescence was evaluated in MACs from 21 low-HDL and 19 high-HDL. Senescent cells were identified using the Senescence Cells Histochemical Staining kit (Sigma-Aldrich Ltd, Poole, Dorset, UK). Briefly, MACs (300,000 cells/well) were washed in PBS, fixed for 7 min at room temperature, washed again and incubated for 16–18 h at 37 °C without CO_2_ and with X-gal chromogenic substrate. After that, cells were washed with PBS, and DMSO was added to dissolve the stain at 37 °C for 30 min; absorbance was measured at 620 nm. To test the effect of an oxidative stress condition on MACs senescence, H_2_O_2_ (1 mM, 1 h) was added to culture medium.

### MACs migration capacity

MACs migration was determined in cells from 25 low-HDL and 23 high-HDL using the 5 µM QCM™ Chemotaxis Assay (Millipore, USA), based on the Boyden chamber principle. After 5 days in culture, the cells were detached using Trypsin/EDTA and harvested by centrifugation; hence, 200,000 cells/well were added to the upper part of a modified Boyden chamber placed in a 24-well culture dish containing EGM-2 and EGM-2 enriched with VEGF (50 ng/mL) (Sigma-Aldrich Ltd, Poole, Dorset, UK) and incubated for 24 h. Cells in the insert were stained and then placed in a well containing a stain extraction buffer after washing in PBS. Migrating cells were counted by colorimetric measurement (optical density at 540 nm).

### ROS production

Reactive oxygen species production was evaluated in cells from 31 low-HDL and 20 high-HDL using ROS-sensitive fluorescent probe 5-(and-6)-chloromethyl-2′,7′-dichloro-di-hydro-fluorescein diacetate, acetyl ester (CM-H_2_DCFDA) (Invitrogen, Life Technologies Ltd). Briefly, MACs (200,000 cells/well) were incubated with CM-H_2_DCFDA (10 µM/well) for 30 min at 37 °C in dark conditions and ROS production was detected as an increase in fluorescence, by a fluorescence microplate reader, at 495 nm excitation and at 527 nm emission.

### Statistical analysis

Statistical analyses were carried out using the SPSS 13.0 software (SPSS Inc., Chicago, I, USA) for Mac OS X. Data are expressed as median (interquartile range) and/or mean ± SD for continuous variables, and number of cases and percentage for categorical variables. Continuous variables were compared by unpaired Student’s *t* test or by one-way ANOVA (with Scheffe post hoc multiple comparisons) for normally distributed variables and by the Wilcoxon Sum-of-Ranks (Mann–Whitney) U test or the Kruskal–Wallis test for variables with skewed distribution. The general linear model (GLM), as an extension of the linear multiple regression for a single dependent variable (each parameter exploring MACs function), has been employed to verify whether HDL levels (the categorical independent factor) still has an effect, beyond the effects of covariates for which significant (or marginally significant) differences have been observed in low-HDL as compared to high-HDL individuals (gender, age, diabetes duration, BMI, waist circumference, non-HDL cholesterol, triacylglycerol, eGFR, presence of hypertension and smoking habits). Estimated marginal means (ESM) have been reported where appropriate. Pearson χ^2^ or the Fisher exact probability tests were applied to categorical variables. Logistic regression analysis with backward stepwise variables elimination was used to assess the independent impact of predictors on each criterion variable stratified by the median level. *p* values of 0.05 or less were considered statistically significant.

## Results

### Characteristics of the study cohort

The main anthropometric and clinical characteristics, and the pharmacological treatments of the study cohort are shown in Table 1. Compared with individuals with low HDL-C those with high HDL-C were more frequently females, were older, and had longer diabetes duration and higher total cholesterol levels. Low-HDL group had higher BMI, waist circumference and prevalence of obesity, higher diastolic BP and prevalence of hypertension, higher non-HDL cholesterol, triacylglycerol, uric acid and creatinine levels, with lower eGFR. Subjects with low-HDL were more frequently current smokers and on blood pressure-lowering treatments. No differences were found for fasting plasma glucose levels, HbA1c, systolic BP, LDL cholesterol, treatment with RAAS (Renin Angiotensin Aldosterone System) inhibitors, lipid-lowering agents or glucose-lowering drugs. Finally, no differences were observed in the prevalence of non-advanced retinopathy, previous major acute cardiovascular events or distribution of UAE categories. MAC studies were conducted in subgroups out of the whole cohort as specified in the “Methods” section.

### MACs functional properties

#### MACs viability

**Fig. 2:**
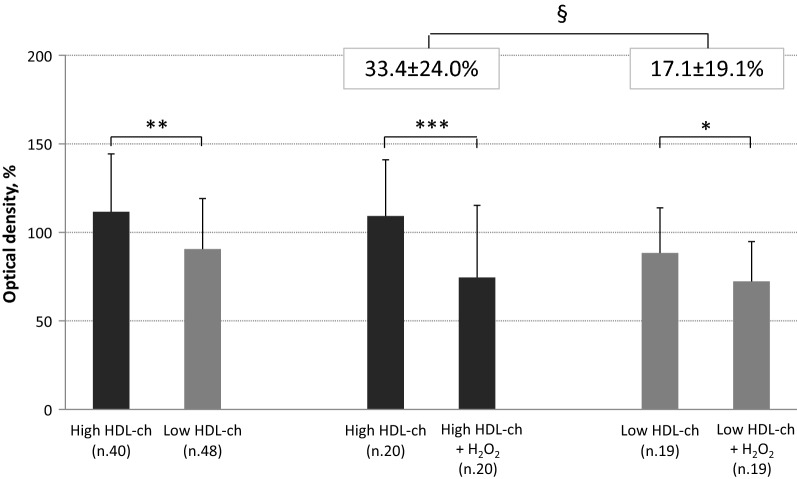
Ex vivo viability of MACs drawn from type 2 diabetic patients with high HDL-cholesterol levels compared to low HDL-cholesterol group (on the left). MACs viability (on the right) was than evaluated in presence or absence of oxidative stress induced by exposure to H_2_O_2_. The bars represent mean ± SD. *p = 0.004; **p = 0.002; ***p < 0.0001 ^§^p < 0.05

Viability of MACs obtained from high-HDL patients was 23% higher than that of cells from low-HDL subjects (111.6 ± 32.7 vs. 90.5 ± 28.6%, p = 0.002). Effect of HDL on viability was still significant (ESM 113.0 ± 45.4 vs. 93.1 ± 42.2%, p = 0.037) beyond the effects of covariates and with an independent role for eGFR (p = 0.047). To investigate high-HDL contribution to MACs protection from oxidative stress, MACs were incubated for 1-h with 1 mM H_2_O_2_. Cell viability decreased in both groups (high-HDL, n. 20: 109.2 ± 31.7% vs. high-HDL + H_2_O_2_ 74.5 ± 40.8%, p < 0.0001; low-HDL, n. 19: 88.3 ± 25.5% vs. low-HDL + H_2_O_2_ 72.3 ± 22.5%, p = 0.004; Fig. 2), with a percent decrease in viability that was significantly higher in MACs from high-HDL than from low-HDL subjects (33.4 ± 24.0% vs. 17.1 ± 19.1%; p = 0.025). Viability measured after exposure to oxidative stress was directly correlated with viability at baseline in the whole sample (r = 0.655, p < 0.0001) as well in high- (r = 0.729, p < 0.0001) and low-HDL groups (r = 0.619, p = 0.005) (Fig. 2).

#### MACs senescence

Ex vivo senescence assess was comparable in the two groups (102.7 ± 29.8% vs. 99.2 ± 27.8%, p = 0.703). In the whole sample, senescence was worsened by exposure to H_2_O_2_ (96.9 ± 24.5% vs. 89.2 ± 23.4%, p = 0.046); but this difference was not statistically significant when high- (98.8 ± 23.3% vs. 92.8 ± 22.7%, p = 0.303) and low-HDL samples (95.2 ± 26.1% vs. 86.1 ± 24.4%, p = 0.088) were evaluated separately. Senescence in response to oxidative stress was directly correlated with senescence at baseline in the whole sample (r = 0.651, p < 0.0001) as well in high- (r = 0.590, p = 0.026) and low-HDL group (r = 0.691, p = 0.003).

#### MACs adhesion capacity to matrix molecules and migration

Ex vivo adhesion to fibronectin did not differ in MACs from subjects with high-HDL (105.2 ± 32.7%) compared to low-HDL (94.1 ± 26.1%, p = 0.223). Also ex vivo migration capacity was similar in MACs from subjects with high-HDL (91.3 ± 34.2%) compared to those with low-HDL (108.7 ± 39.5%, p = 0.111).

#### ROS production

Reactive oxygen species production did not differ in MACs from individuals with high-HDL (94.5 ± 30.4%) compared to those with low-HDL (103.6 ± 32.0%, p = 0.316).

### MACs functional properties by HDL-cholesterol quartiles

**Fig. 3:**
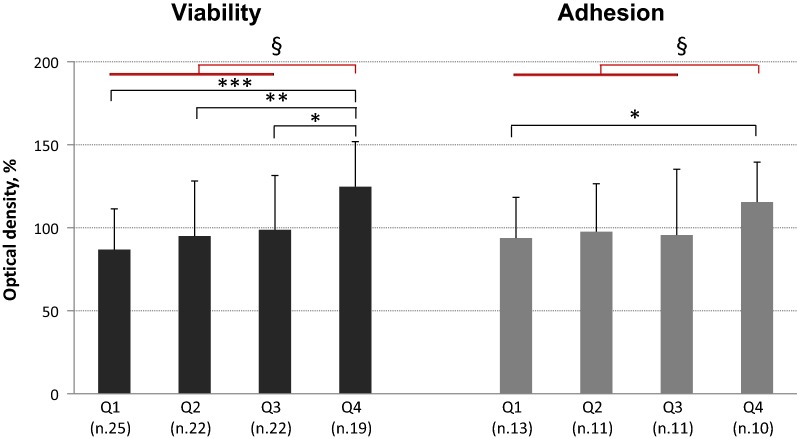
Ex vivo viability and adhesion of MACs drawn from type 2 diabetic patients stratified by HDL-cholesterol level quartiles: Q1 < 34 mg/dl; Q2 34–42 mg/dl; Q3 42–71 mg/dl; Q4 > 71 mg/dl. The bars represent mean ± SD. Viability, on the left (ANOVA one-way p = 0.001): *p = 0.055; **p = 0.020; ***p = 0.001; ^§^p < 0.0001. Adhesion, on the right: *p = 0.072; ^§^p < 0.04

To further evaluate the relationship between HDL-C levels and MACs functional properties, the study population was stratified into quartiles (Q) of HDL-C levels yielding quartiles thresholds of 34, 42 and 71 mg/dl, respectively (0.88, 1.09 and 1.84 mmol/l). Viability increased from Q1 to Q4 (p = 0.001) and was significantly higher in Q4 (124.8 ± 27.1%) than in Q1 (86.9 ± 24.5%, p < 0.001), Q2 (95.0 ± 33.2%, p = 0.020) and, marginally, than in Q3 (98.8 ± 32.7%, p = 0.055); this remained by comparing Q4 and Q1–Q3 (124.8 ± 27.1 vs. 93.3 ± 30.1%, p < 0.0001, Fig. 3). Effect of HDL quartiles on viability was still significant (p = 0.008) beyond the effects of covariates and with a marginal role for eGFR (p = 0.094). ESM was 128.6 ± 39.3 in Q4 vs. 88.0 ± 36.6 (p < 0.001), 93.2 ± 35.7 (p = 0.006) and 97.1 ± 39.2% (p = 0.004) in Q1–Q3, respectively. This difference persisted comparing Q4 and Q1–Q3 (126.7 ± 36.9 vs. 92.4 ± 42.7%, p < 0.001), with marginal effects for both eGFR (p = 0.092) and diabetes duration (p = 0.090). Consistently, adhesion was higher in Q4 than in Q1–Q3 (115.5 ± 24.1 vs. 95.6 ± 30.4%, p = 0.044; Fig. 3). This remained marginally significant (ESM 114.4 ± 32.0 vs. 96.0 ± 30.6%, p = 0.057) beyond the effects of covariates. No differences by HDL-C quartiles were observed for senescence, migration or ROS production.

### Independent covariates of MACs functional properties

HbA1c and HDL-C resulted independent covariates of MACs viability: indeed, viability increased with the improvement of HbA1c (OR 0.789) as well with the increase in HDL-C (OR 1.034) (Table 2).

**Table 2:** Independent correlates of MACs functional properties (logistic regression analysis with stepwise backward variables elimination)

	Viability			Adhesion			Senescence		
	OR	95% CI	*p*	OR	95% CI	*p*	OR	95% CI	*p*
BMI (× 1 kg/m^2^)	–	–	–	0.949	0.908–0.991	0.019	0.878	0.779–0.990	0.033
HbA1c (× 1%)	0.789	0.678–0.919	0.002	–	–	–	1.683	1.043–2.717	0.033
HDL-cholesterol (× 1 mg/dl)	1.034	1.013–1.055	0.001	1.029	1.005–1.053	0.018	–	–	–
Other variables included in the model, but never selected as significant independent covariates	Sex, age, waist circumference, diabetes duration, fasting glucose, LDL cholesterol, triacylglycerol, systolic and diastolic BP, eGFR, albumin-to-creatinine ratio, smoking habits, treatments with BP-lowering and lipid-lowering agents								

When HDL quartiles were included in the regression instead of HDL-C levels, Q4 was strongly associated with EPCs viability (OR 8.343; 95% CI 2.219–31.369, p = 0.002).

Independent predictors of the adhesion capacity were HDL-C and BMI, i.e. adhesion was directly related to HDL levels (OR 1.029) and inversely with BMI (OR 0.949). When HDL quartiles were included in the regression instead of HDL-C levels, Q4 was strongly associated with MACs adhesion capacity (OR 6.492; 95% CI 1.217–31.635, p = 0.011). Finally, HbA1c (OR 1.683, directly) and BMI (OR 0.878, inversely) emerged as independent correlates of MACs senescence.

## Discussion

This study shows that in subjects with type 2 diabetes HDL-cholesterol levels can affect MACs functions. In particular, ex vivo MACs from patients with high-HDL have higher viability and greater adhesion capacity to matrix molecules compared to MACs from individuals with low-HDL while no difference was apparent with respect to senescence, migration capacity, and ROS production. Finally, we did not observed any concentration-related biphasic effects of HDL as previously suggested [14].

MACs, originally defined as “early EPCs” or “early outgrowth EPCs” [28], have been obtained, as recently reviewed [29], by short-term culturing of PBMCs. Growth occurs after a few days of adhesion to fibronectin in VEGF-containing medium. This technique yields cells with myeloid/hematopoietic characteristics that share features particularly with monocyte/macrophages. MACs, a terminology that clarify both lineage and function of these cells, are able to promote angiogenesis in vivo not necessarily by means of an endothelial commitment, although co-expression of endothelial markers by these cells have been widely reported [28] and herein confirmed. Indeed, cells gained by short-term culture procedures likely enhance vessel formation predominantly by a paracrine mechanism via a mixture of growth factors and cytokines that support angiogenesis, rather than becoming integrated as long-lived endothelial cells or directly contributing to re-endothelialization process. Nevertheless, MACs (“early EPCs”), that are not endothelial nor progenitor cells, albeit cells with pro-angiogenic vasoreparative properties [29], have been suggested as putative biomarkers for cardiovascular disease. Thus, ex vivo assessment of MACs function is especially important as these cells are recognized for their role in vascular repair in health and disease and have been harnessed as therapeutic tools for many ischemic diseases.

Our results can shed some light on the cardiovascular protection HDL-cholesterol may exert in type 2 diabetic subjects. Low levels of HDLs are a typical component of diabetic dyslipidemia [30]. Furthermore, even in those subjects with normal or higher HDL-cholesterol levels, modification of the lipoprotein can hamper their anti-atherogenic properties [30, 31] including the positive effect on bioavailability and functional properties of EPCs [3, 32]. This effect is supported by experimental evidence. In a mice model i.v. injection of rHDLs increased the number of bone-marrow-derived endothelial cells in the ischemic muscle [33] and their recruitment into the murine aortic endothelial layer in response to an inflammatory insult [11]. In patients with type 2 diabetes, rHDLs infusion increases plasma HDL anti-inflammatory properties, enhances ex vivo cholesterol efflux capacity [34], and restores endothelial function as determined by the forearm blood flow response to serotonine [21] as well as the number of circulating EPCs [35]. The increased number of circulating EPCs as a result of mobilization of steam/progenitor cells from the bone marrow to the peripheral blood in response to HDL infusion has been demonstrated in vivo in mouse models and in humans [11, 33, 35]. On the contrary, the hypothesis of an improved viability and survival suggested by our results was only shown using ex vivo assays. This last hypothesis is indeed in agreement with the greater ex vivo viability of MACs from type 2 diabetic individuals with high HDL-C levels (Fig. 2) and is further supported by the dose–response relationship between quartiles of HDL-C and MACs viability (r = 0.396, p < 0.001; Fig. 3).

In the past, a dual effect of HDLs on EPCs has been reported as a function of HDL concentration with enhanced EPCs tube formation in the presence of low levels and a paradoxical increase of senescence and impaired tube formation at moderate to high concentration [14]. Though we did not see such a clear-cut effect, it is of interest that the relationship between HDL-C levels and ex vivo MACs adhesion to matrix molecules is enhanced only in the highest HDL-C quartile (Fig. 3).

Our results show that HDL-C has no effects on ex vivo MACs senescence and migration capacity as well on ROS production. The antioxidant capacity of HDLs is mainly conferred by apolipoproteins and related enzymes such as paraoxonases (PON), platelet-activating factor-acetyl hydrolase (PAF-AH), glutathione peroxidase (GPx) and others [3]. In endothelial cells, HDLs can inhibit intracellular oxidative stress through inactivation of NADPH oxidase [3]. In our hands, in MACs from patients with high HDL-C levels, intracellular ROS generation was not lower as compared to those from low HDL most likely because of impaired anti-inflammatory and antioxidant capacity of HDL associated with the lipoprotein modifications occurring in diabetes [36, 37]. MACs are uniquely equipped with intrinsic cellular machinery for ROS detoxification. As such, they are more resistant to oxidative stress as compared with mature endothelial cells [26, 38]. However, impaired EPCs viability and function as well as increased apoptosis have been observed in EPCs under conditions of oxidative stress as generated by hydrogen peroxide [38–40]. Consistently, exposure of MACs obtained from subjects with type 2 diabetes to H_2_O_2_ reduced their ex vivo viability (Fig. 2) and accelerated senescence irrespective of HDL-C concentrations suggesting those HDL particles may become ineffective when challenged by oxidative stress. In support to this hypothesis is the finding that percent reduction viability in response to H_2_O_2_ (Fig. 2) was higher in MACs from type 2 diabetes individuals with high HDL-C in spite of greater viability on basal condition. A similar mechanism may also account for the lack of difference in ex vivo EPCs senescence ad migration as previously shown [41].

A number of mechanisms may support HDL incompetence in diabetes including metabolic alterations typically associated with this condition. In line with this interpretation we found that ex vivo MACs viability is directly related to HDL-C levels and inversely associated with HbA1c, but not with diabetes duration. This observation is in keeping with results reported by Tepper et al. [32] showing that proliferation of EPCs from type 2 diabetic subjects is inversely correlated with HbA1c, while EPC-bearing clusters inversely correlated with duration of diabetes.

We also found an inverse correlation between the MACs adhesion capacity and BMI. This association is not surprising given the well known effect of body weight on circulating EPCs [42, 43], an effect that seems to be even greater than the one exerted by blood pressure, LDL cholesterol, triacylglycerol, fasting glucose, and smoking [43]. In the study by Heida et al. [44], EPCs expanded from obese normo-glycemic subjects exhibited reduced adhesive, migratory, and angiogenic capacity, and mice treated with obese-derived EPCs showed reduced homing in ischemic hind limbs. Interestingly, functional impairment of EPCs was reversible after achieving significant weight reduction [44]. In summary, our study explores function of ex vivo MACs and emphasizes the role of body weight and glycemic control on their senescence.

Several other factors can, obviously, affect MACs function in diabetes. For instance, the presence, type, and degree of diabetic complications have been reported to be associated with a whole array of numerical and/or functional impairments of EPCs [45]. Decreased as well as increased or unchanged EPC number has been reported in diabetic patients with severe retinopathy. Of note, where increased number of circulating EPCs has been reported in patients with diabetic retinopathy, EPC functions such as migration and mobilization or homing were often impaired [18, 45]. Moreover, diabetic complication such as CKD can deeply affect HDLs particle number and function. For all these reason, we have paid attention not to include subjects with advanced diabetic retinopathy and those with CKD stages ≥ 3b in this study. Nevertheless, also in these conditions, an independent effect of reduced eGFR seems to contribute to impaired MACs viability. Some blood-pressure lowering drugs (RAAS blockers), lipid-lowering agents (statins) and anti-hyperglycemic treatments [45] also may have an effect of MACs. However, in our study, all these treatments were evenly distributed in type 2 diabetic subjects with low- and high-HDL cholesterol levels. Moreover, treatments did not enter as independent covariates of MACs functional properties.

Because of all this, we are confident that HDL levels largely mediate the effects we have studied. With respect to this, we have to acknowledge that other features of these lipoproteins such as their size, composition, and function may have played a role. Epidemiological studies, for instance, suggest that large, buoyant HDL particles (i.e. HDL_2_) may be a better marker of favorable cardiovascular outcomes [46, 47] though it has been recently suggested that HDL_2_ and HDL_3_ cholesterol do not necessarily distinguish cardioprotective effects of HDL subclasses [48].

Some limitations of our study must be taken into consideration. First, data on circulating EPCs levels are not available. This could have provided a complementary information on the link between HDL and MACs alterations in type 2 diabetes. Indeed, studies have reported that HDL cholesterol levels correlate with the number of circulating CD34+/KDR+ EPCs in subjects with coronary artery disease [5], and with the number of CD34+/CD133+ EPCs in hypercholesterolemic subjects [6] and in obese non diabetic women [49]. Furthermore, EPC colony levels were significantly lower in individuals with low HDL in a cohort of patients with cerebrovascular disease including a subgroup of diabetic subjects [9]. In a cohort of volunteers with different degrees of glucose tolerance we have previously reported a significant correlation between HDL-cholesterol and circulating CD34+ cells but not with CD34+/KDR+ cells [50]. Thus, to the best of our knowledge, the impact of HDL concentrations on circulating EPC levels in diabetes remains a poorly explored field.

Second, ex vivo HDL treatment could have provided further evidence in support of the role for HDL in MAC dysfunction. Literature data show that intravenous infusion of rHDLs stimulates EPC differentiation and recruitment in rodents [11, 33], exerts beneficial effects on circulating CD34+ cells in patients with recent acute coronary syndrome [51] and increases circulating CD34+/VEGFR2+ cells in patients with type 2 diabetes [35]. Of interest, a long-term trial with Mediterranean diet, that reported among other effects a sustained increase in HDL cholesterol levels, showed a long-term increase in circulating EPCs levels in patients with newly diagnosed type 2 diabetes [52].

Another study limitation relates to the lack of in vivo assessment of cell function. Previous studies have shown that administration of cultured MACs (“early” EPCs) obtained from diabetic subjects to mice did not promote reendothelialization at site of endothelial injuries nor did restore perfusion in ischemic tissues to the same extent of what obtained with cells of healthy controls [53]. However, it is unknown whether MACs derived from diabetic subjects with high HDL cholesterol might translate their preserved capability in promoting the formation of functional vascular networks in vivo.

Finally, we must recognize the lack of mechanistic data on how HDL would impact EPC function in diabetes. An increasing number of experiments shows that diabetes impairs the stromal-derived factor-1 (SDF-1)/C-X-C chemokine receptor type 4 (CXCR-4) and the nitric oxide (NO)/superoxide anions pathways and the p53/sirtuin1 (SIRT1)/p66Shc axis. All these are major regulators of cell proliferation, migration and reparative properties of diabetic cultured myeloid and circulating putative EPCs [53]. Furthermore, in circulating angiogenic cells isolated from peripheral blood of diabetic subjects it was shown that the downregulation of several microRNAs (microRNA-155, -126 and -130a) contributes to reduce their proliferation, promote their senescence and apoptosis, and impair their reparative functions. Apart from microRNAs, other epigenetic mechanisms, including damages and post-translational modifications of DNA, are known to be dysfunctional in diabetes [54], but, to our knowledge, none of these putative mechanisms has been investigated in relation to the serum concentration of HDL. Instead, it is widely recognized that overall HDL in individuals with type 2 diabetes wastes the capacity to suppress NF-kB-mediated inflammatory response and loses the ability to stimulate eNOS activation [55].

In particular, it could have been of interest to explore the role of apolipoprotein A-I (apoA-I) and angiopoietin-like protein 3 (ANGPTL3), a major lipoprotein regulator that shows positive correlation with plasma HDL cholesterol and apoA-I levels. To this regard, it has been recently reported that ANGPLT3 levels are lower in female T2DM patients with a weaker association with HDL components (apoA-I and serum amyloid A) and function (cholesterol efflux) [56]. ANGLPT3 might also play a role in angiogenesis. Similarly, it could be of value to assess the expression of the scavenger receptor type BI (SR-BI), a HDL receptor whose deficiency is associated with impaired HDL function, intracellular cholesterol accumulation, increased oxidative stress and regulates hematopoietic stem/progenitor cells proliferation and differentiation [57].

## Conclusions

In conclusion, we suggested that MACs derived from type 2 diabetic individuals with high HDL-C levels show a relative preservation of some functional properties (mainly viability, to a lesser extent adhesion) as evaluated ex vivo as compared with cells obtained from subjects with low HDL-C. Except for viability and adhesion, other MACs functions were only marginally or not at all related to HDL levels. These properties may be important in protecting vascular wall integrity.

## Abbreviations

AcOH: acetic acid
ACR: albumin-to-creatinine ratio
ALT: alanine aminotransferase
apoA-1: apolipoprotein A-1
AST: aspartate aminotransferase
BMI: body mass index
BP: blood pressure
CKD-EPI: chronic kidney disease epidemiology collaboration
CM-H2DCFDA: 5-(and-6)-chloromethyl-2′,7′-dichloro-di-hydro-fluorescein diacetate, acetyl ester
CVD: cardiovascular disease
DiI-ac-LDL: 1,1′-dioctadecyl-3,3,3′,3′-tetramethylindocarbocyanine-labeled acetylated low-density lipoprotein
DMSO: dimethyl sulfoxide
EBM-2: endothelial basal medium-2
e-CFU: endothelial colony-forming unit
EDTA: ethylenediaminetetraacetic acid
EGF: endothelial growth factor
eGFR: estimated glomerular filtration rate
EGM-2-MV: microvascular Endothelial Cell Growth Medium**-**2
EMPs: endothelial cell-derived microparticles
EPCs: endothelial progenitor cells
ESM: estimated marginal means
FBS: fetal bovine serum
FGF: fibroblast growth factor
FITC: fluorescein isothiocyanate
FMD: flow-mediated vasodilation
GGT: gamma-glutamyltransferase
GLM: general linear model
GPx: glutathione peroxidase
H_2_O_2_: hydrogen peroxide
HbA1c: hemoglobin A1c
HDLs: high-density lipoproteins
HDL-C: HDL-cholesterol
IGF-1: insulin-like growth factor-1
LDL: low-density lipoprotein
MACs: monocytic angiogenic cells
MTT: 3-(4,5-Dimethylthiazol-2-yl)-2,5-diphenyltetrazolium bromide
NO: nitric oxide
OECs: outgrowth endothelial cells
oxLDL: oxidized LDL
PBMCs: peripheral blood mononuclear cells
PBS: phosphate buffered saline
PE: phycoerythrin
PAF-AH: platelet-activating factor-acetyl hydrolase
PON-1: paraoxonase-1
RAAS: renin–angiotensin–aldosterone system
rHDL: reconstituted HDL
ROS: reactive oxygen species
UAE: urinary albumin excretion
VEGF: vascular endothelial growth factor
VEGFR-2: vascular endothelial growth factor receptor-2
VPT: vibration perception threshold
X-gal: 5-Bromo-4-chloro-3-indolyl ß-d-galactopyranoside
